# Performance and Safety of the Medical Device Ialuxid Gel in the Treatment of Mild–Moderate Acne Vulgaris: An Open‐Label, Noncomparative Multicentre Interventional Clinical Trial

**DOI:** 10.1111/jocd.70084

**Published:** 2025-03-03

**Authors:** Meda‐Elena Stefancu, Dionisio Franco Barattini, Ionel Botnaru, Carmen Vizman, Luca Stucchi, Luca Barattini

**Affiliations:** ^1^ S.C. Salvosan Ciobanca S.R.L. Zalău Jud Salaj Romania; ^2^ Opera CRO, A TIGERMED Company Timisoara Romania; ^3^ Darzas Aesthetic Oradea Romania; ^4^ Nova‐Clin Medical Research Center S.R.L. Timisoara Romania; ^5^ BMG Pharma SpA Milano Italy; ^6^ TIGERMED Italy Srl IT Department Genova Italy

**Keywords:** acne vulgaris, hyaluronic acid, hydrogen peroxide, performance, safety

## Abstract

**Background:**

Medical literature shows there is no ideal treatment for acne, but topical therapies like benzoyl peroxide, retinoids, and antibiotics have proven to improve mild–moderate cases. Replacing benzoyl peroxide (potentially irritating) with hydrogen peroxide has already been suggested in the medical literature.

**Aims:**

We investigated a medical device, a proprietary combination of hyaluronic acid, hydrogen peroxide, and glycine in mild–moderate acne vulgaris.

**Methods:**

Patients of both sexes between ≥ 18 and ≤ 45 years old, with a Global Acne Grading System score ≤ 30, were included. Exclusion criteria were dermal systemic or infectious diseases; allergy to the tested product; pregnant or lactating women; treatment for acne 30 days before baseline; and previous facial aesthetic surgery. Forty patients were treated for 8 weeks several times a day with the tested product and visited at baseline, week 2, week 4, and week 8. Outcomes were decreasing the total number of lesions and the Global Acne Grading System Severity Score, Investigator Global Assessment of Performance with photographs evaluation in blind, Dermatology Life Quality Index, and the Treatment Satisfaction Questionnaire. Adverse events were collected for safety.

**Results:**

A 2‐fold decrease in the number of lesions at the final visit (−56.3%, *p* < 0.001). The remaining outcomes evidenced a statistically significant reduction at the final visit. Only 8 adverse events (all mild and related to the tested device) were reported.

**Conclusions:**

Results and optimal safety demonstrate that the tested product has a clinical benefit and could be associated with retinoids, as first choice for mild to moderate acne.

**Trial Registration:**

Clinicaltrial.gov as NCT05345093

**Abbreviations**
ADE: Adverse Device Event
AE: Adverse Event
ANCOVA: Analysis of Covariance
ANOVA: Analysis of Variance
BPO: Benzoyl peroxide
DD: Device Deficiency
DLQI: Dermatology Life Quality Index
EC: Ethical Committee
GAA: Global Alliance Acne
GAGS: Global Acne Grading System
GCP: Good Clinical Practice
GDPR: General Data Protection Regulation
HA: Hyaluronic Acid
HPO: Hydrogen peroxide
ICH: International Conference on Harmonization
IFU: Instructions for Use
IGAP: Investigator Global Assessment of Performance
IGAS: Investigator Global Assessment of Safety
IMD: Investigational Medical Device
ITT: Intention to treat
MDIC: Medical Device based on Ialuvance Complex
NCMMD: National Commission for Bioethics of Medicines and Medical Devices
PGAS: Patient Global Assessment of Safety
PMCF: Post‐Market Clinical Follow‐up
PP: per protocol
SAE: serious adverse event
SD: standard deviation
TSS: Total Severity Score

## 1 Background

The skin, the largest organ in the human body, serves as a barrier against chemical and biological hazards. The visible appearance of the skin can reveal health status, and certain skin conditions, even nonlife threatening ones, can lead to severe psychological effects on those affected [1]. Indeed, skin diseases are a major health concern; they are caused by both intrinsic and extrinsic factors, among which are bacterial infections [2]. Diagnosing skin bacterial infections is based on skin appearance and bacterial identification. Common *Staphylococcus* and *Streptococcus* strains are most common, with *Staphylococcus aureus* being the most dangerous due to its potential for bacteraemia, infecting heart valves and bones, potentially causing endocarditis and osteomyelitis. Skin infections due to *Staphylococcus aureus* can include folliculitis, impetigo, cellulitis, and abscesses [3].

Acne vulgaris is characterized by the formation of comedones and sebaceous plugs impacted within follicles, characterizing noninflammatory acne, and by the formation of papules, pustules, nodules, and/or cysts as a result of obstruction and inflammation of pilosebaceous units (hair follicles and their accompanying sebaceous glands), characterizing inflammatory acne. It occurs through the interplay of four major factors: excess sebum production, follicular plugging with sebum and keratinocytes, colonization of follicles by *Cutibacterium acnes* (formerly known as *Propionibacterium acnes* ), and the release of multiple inflammatory mediators. Indeed, acne vulgaris is now approached as an inflammatory condition, focusing on androgens, hormone receptors, regulatory neuropeptides, and environmental factors as agents able to interfere with the natural cyclical dynamic breakdown of devitalized cells into sebum inside the sebaceous follicles. Blockage of the discharge of sebum to the surface of the skin leads to obstruction of the ducts (microcomedones) and then bigger comedones that become inflammatory lesions [4].

The current medical literature shows that there is no ideal treatment for acne, but topical therapies like benzoyl peroxide (BPO), retinoids, and antibiotics have proven to be able to improve mild to moderate acne control [5, 6]. In the algorithm presented by the Global Alliance Acne (GAA) [7] in 2009, BPO associated with retinoids is indicated as the first choice in the treatment of mild and moderate papulopustular acne. The GAA confirmed this relevant role of BPO in a recent paper on the practical management of acne addressed to clinicians [8]. This guideline, partially modifying previous indications, strongly does not recommend the monotherapy with topical or systemic antibiotics and, at the same time, indicates BPO alone or in fixed combination with retinoid (i.e., adapalene) as the preferred topical antimicrobial agent due to the current climate of antimicrobial stewardship. BPO is on the market as an anti‐acne product since 1990 and several cases (also severe) of skin irritation and dryness have been reported [9]. For these reasons, the substitution of BPO with hydrogen peroxide (HPO) was proposed. The clinical trials [10, 11] that compared the two formulations in the treatment of mild and moderate papulopustular acne (following the classification reported in the 2016 European S3 Acne Guideline) [12] evidenced the same efficacy. These results versus BPO were also confirmed using formulations of HPO in association with adaptalene [13] or salycilate acid and D‐Panthenol [14, 15]. Consequently, there is a growing interest in finding ingredients that can be combined with HPO to create a dermatologically safe and effective product for the treatment of acne. To address this need, BMG Pharma S.p.A. (Milan, Italy) has developed the medical device Ialuxid (MDIC), a gel based on the Ialuvance complex, a proprietary combination of hyaluronic acid (HA), HPO, and glycine.

## 2 Aim of the Study

The aim of this clinical trial was to investigate whether 8 weeks of treatment with MDIC gel according to the instructions for use (IFU) could improve the course of the disease in a population affected by mild–moderate acne vulgaris.

## 3 Subjects and Methods

### 3.1 Study Design

This multicenter open‐label, single‐group assignment interventional Postmarket Clinical Follow‐up (PMCF) study with MDIC gel was performed at three sites located in Romania.

### 3.2 Ethics

The study protocol was initially approved by the National Commission for Bioethics of Medicines and Medical Devices (NCMMD) on 6 October 2022 (sites 01 and 02) and 11 May 2023 (site 03). The amendment with the final version of the protocol dated 1 April 2023 was approved by the NCMMD on 25 October 2023.

The trial was conducted in accordance with the Declaration of Helsinki and complied with the International Conference on Harmonization (ICH), Good Clinical Practice (GCP), Medical Devices guidelines (MEDDEV), ISO 14155, and Romanian regulatory requirements. It was registered on Clinicaltrial.gov as NCT05345093, and written informed consent forms (previously approved by NCMMD) were obtained from all participants for their participation in the trial and for data treatment (according to General Data Protection Regulation (GDPR) 2016/679).

To ensure the quality of the data collected, an independent Contract Research Organization (CRO), Opera CRO (Timisoara, Romania), was selected to manage the logistics and conduct the study (protocol submission, project management, site monitoring, data management and statistical analysis).

### 3.3 Subjects

The subjects enrolled in the study were outpatients who had a visit for acne in the following private clinics in Romania specialized in dermatology: SC Salvosan Ciobanca in Zalău (site 01), Darzas Esthetic in Oradea (site 02) and Nova‐Clin Medical Research Center in Timişoara (site 03). The trial included patients of both sexes aged ≥ 18 and ≤ 45 years, diagnosed with mild to moderate acne vulgaris with Global Acne Grading System (GAGS) score ≤ 30, agreeing to discontinue any other dermatological treatments and procedures during the trial. The main exclusion criteria were: patients with any dermal systemic pathologies or suffering from infectious diseases; patients with a history of anaphylaxis, allergy, or hypersensitivity to HA or to any other ingredients of the tested product; patients unlikely to cooperate, pregnant or lactating women; participation in any other interventional trial in the 30 days prior to the study. In addition, patients who used oral drugs or topical therapies for acne vulgaris in the month before the study, or who underwent facial esthetic surgery or facial rejuvenation surgery (including filler injections and botulinum toxin A) were also excluded. The concomitant use of quaternary ammonium salts was not allowed for the well‐known incompatibility between HA and drugs such as benzalkonium chloride.

### 3.4 Study Visits

Each patient was evaluated for eligibility to enter the trial at a screening visit. After the examinations performed at baseline (day 0), the subject was visited at weeks 2, 4, and 8. Subjects were informed that the Investigator should be contacted in the event of an adverse event (AE) and that in the case of a severe AE an unscheduled visit could be immediately performed. Table 1 outlines the schedule of examinations and procedures to be performed during the study period.

**TABLE 1:** Demographic characteristics and vital signs measurements at baseline visit.

Characteristics		Overall	Female	Male	*p* *
Age (years)	*N*	36	29	7	0.035^1^
	Mean (SD)	25.9 (6.27)	26.9 (6.12)	21.4 (5.09)	
	Median	25.5	26	19	
	Range	[18–40]	[18–40]	[18–31]	
Weight (kg)	*N*	36	29	7	NR
	Mean (SD)	66.7 (15.0)	64.0 (13.2)	78.1 (17.8)	
	Median	64.8	62	75	
	Range	[45–110]	[45–110]	[54.7–105]	
Height (cm)	*N*	36	29	7	NR
	Mean (SD)	168.1 (9.74)	165.3 (7.23)	179.6 (10.88)	
	Median	168	167	185	
	Range	[151–192]	[151–177]	[164–192]	
Body Mass Index (kg/m^2^)	*N*	36	29	7	0.603^2^
	Mean (SD)	23.56 (4.6)	23.43 (4.76)	24.09 (4.15)	
	Median	22.52	22.49	22.55	
	Range	[16.56–39.44]	[16.56–39.44]	[18.99–28.63]	
Systolic Blood Pressure (mmHg)	*N*	36	29	7	0.275^2^
	Mean (SD)	114.6 (13.03)	113.6 (14.04)	118.6 (6.90)	
	Median	115	113	120	
	Range	[60–140]	[60–140]	[110–130]	
Diastolic Blood Pressure (mmHg)	*N*	36	29	7	0.436^2^
	Mean (SD)	72.8 (9.18)	72.4 (9.94)	74.6 (5.13)	
	Median	71.5	71	72	
	Range	[60–110]	[60–110]	[70–80]	
Heart rate (bpm)	*N*	36	29	7	0.532^2^
	Mean (SD)	73.4 (7.76)	73.9 (8.35)	71.3 (4.39)	
	Median	71	72	70	
	Range	[57–97]	[57–97]	[65–78]	

Abbreviation: NR, Not Relevant.

* *p*‐values by ANOVA^1^ or Mann–Whitney^2^ tests.

### 3.5 Tested Product and Concomitant Treatment

The tested product MDIC gel is a medical device already on the market in several countries. Its ingredients are HA, HPO, and glycine. HA is a critical component of the extracellular matrix, involved in numerous physiological processes, and it plays a vital role in every phase of tissue repair. Its physicochemical and biological properties make it effective in wound repair and the treatment of connective tissue disorders. HA also provides soothing relief for skin issues like dryness and itching [16, 17]. The repairing action of HA on connective tissue is potentiated by the concomitant activity of HPO, which favors tissue regeneration [11], and glycine, which accelerates cutaneous barrier repair [18]. In vivo studies [19] were performed on healthy volunteers to test the anti‐aging potential of glycine. In particular, a decrease in skin roughness parameters (although not statistically significant) and a significant reduction in lipoperoxidation in the stratum corneum were observed. In addition, glycine's action in strengthening the skin's barrier function was demonstrated by a decrease in transepidermal water loss. There is currently no study that has evaluated the effective role of glycine in the Ialuvance complex of MDIC. For this reason, glycine is listed as a buffer in the IFU of the product.

When MDIC is distributed on the affected area, the ingredients create a protective barrier against external agents, maintain a moist environment conducive to healing, and help prevent bacterial infections. For these reasons, the MDIC gel is suitable for acne lesions, folliculitis, paronychia, and Molluscum contagiosum. During the study, MDIC gel was self‐administered by the patients at home for 56 consecutive days, and the dosage was dependent on the skin condition and the extension of treated areas (as indicated in the IFU). The subject should apply the amount necessary to cover the lesion until evenly distributed. The administrations were several times a day when needed. During the study duration, the patients were not allowed treatments to use any product listed in the exclusion criteria section. No restriction on treatments taken previously by patients for clinical conditions not related to the studies conditions was applied.

### 3.6 Study Outcomes

The primary outcome was the decrease in the total number of lesions present on the body area mainly interested by the disease at 8 weeks after the initiation of treatment, compared to day 0. Secondary outcomes objectively evaluated at the day 28 and 56 visit by Investigator were the following. Global Acne Grading System (GAGS) [4] score, which severity was graded as mild = 1–18, moderate = 19–30, severe = 31–38, and very severe > 38. Total Severity Score (TSS) calculated by summing the intensity for each specific sign of the disease, sign intensity graded according to a 4‐point scale, from “absence” to “severe” Investigator Global Assessment of Performance (IGAP) assessed by means of photographs taken by the Investigator at each visit of the study. A second Investigator, blinded to the identity of the patients and the chronological order of the photographs, evaluated the pictures in accordance with best practices. Written informed consent for digital imaging was obtained from each potential patient before the beginning of the study, specifying that the pictures would be used in a clinical trial. A second informed consent form was presented to patients to use their anonymized images by covering the patient's eyes and, if necessary, cropping the untreated facial areas (e.g., chin, lips) for publication in a scientific journal. While the first consent was mandatory for inclusion in the study, the second was optional, and only a very limited number of subjects agreed to sign it. The clinical setting and procedures for taking the photographs, including lighting and camera angles, were detailed in an appendix to the study protocol (Data S1). Photographs were graded on a 4‐point scale from “very good” to “poor” performance.

Additional secondary outcomes were assessed at day 56 by the patient by means of the Dermatology Life Quality Index (DLQI) [20, 21] (scores between 0 and 1 = “no effect at all on patient's life”; 2–5 = “small effect on patient's life”; 6–10 = “moderate effect on patient's life”; 11–20 = “very large effect on patient's life”; 21–30 = “extremely large effect on patient's life”) and by the Treatment Satisfaction Questionnaire.

The safety of the tested medical device was evaluated by means of the assessment of any Adverse Event/Adverse Device Effect (AE/ADE) and Device Deficiency (DD) occurred during the study and the Investigator and Patient Global Assessments of Safety (IGAS and PGAS).

### 3.7 Sample Size

To calculate the necessary sample size, a trial previously performed in Korea [22] was used as a reference. By performing a paired t‐test with a significance level of 0.05 and a power of 0.85, a medium effect size of 0.52 (a smaller effect size than that used in the study above mentioned) was used. Consequently, a sample size of 36 evaluable patients should have sufficed in demonstrating tested product performance under the above‐mentioned statistical test parameters. After considering a possible dropout rate of 10% during the clinical trial, a total of 40 patients affected by acne that should have been enrolled for participation in the clinical study was reached.

### 3.8 Statistical Analysis

Quantitative variables (i.e., demographic), if normally distributed, were described through media, standard deviation (SD); variables nonnormally distributed were described using median and range of interquartile. The Student's t‐test for paired data and the Wilcoxon signed rank test were used to perform comparative analysis in accordance with the distribution of these variables. Categorical variables were described using frequencies and percentages and comparative analysis used the χ2 test or Fisher's exact test. Additionally, ANOVA for repeated measures and/or Linear Mixed Models analyses were performed to assess the secondary endpoints.

The primary endpoint, the change in total lesions count from baseline to the day 56 visit, was analyzed by performing a Student's t‐test for paired data or a Wilcoxon signed rank test if major deviations from the former's test assumptions were recorded.

Secondary endpoints, all continuous variables in nature, were assessed by performing ANOVA for repeated measures tests or ANCOVA for repeated measures tests (if considering covariates like age, sex, body mass index, etc.), assuming completeness of data (no missing data).

## 4 Results

### 4.1 Patients Disposition

**FIGURE 1:**
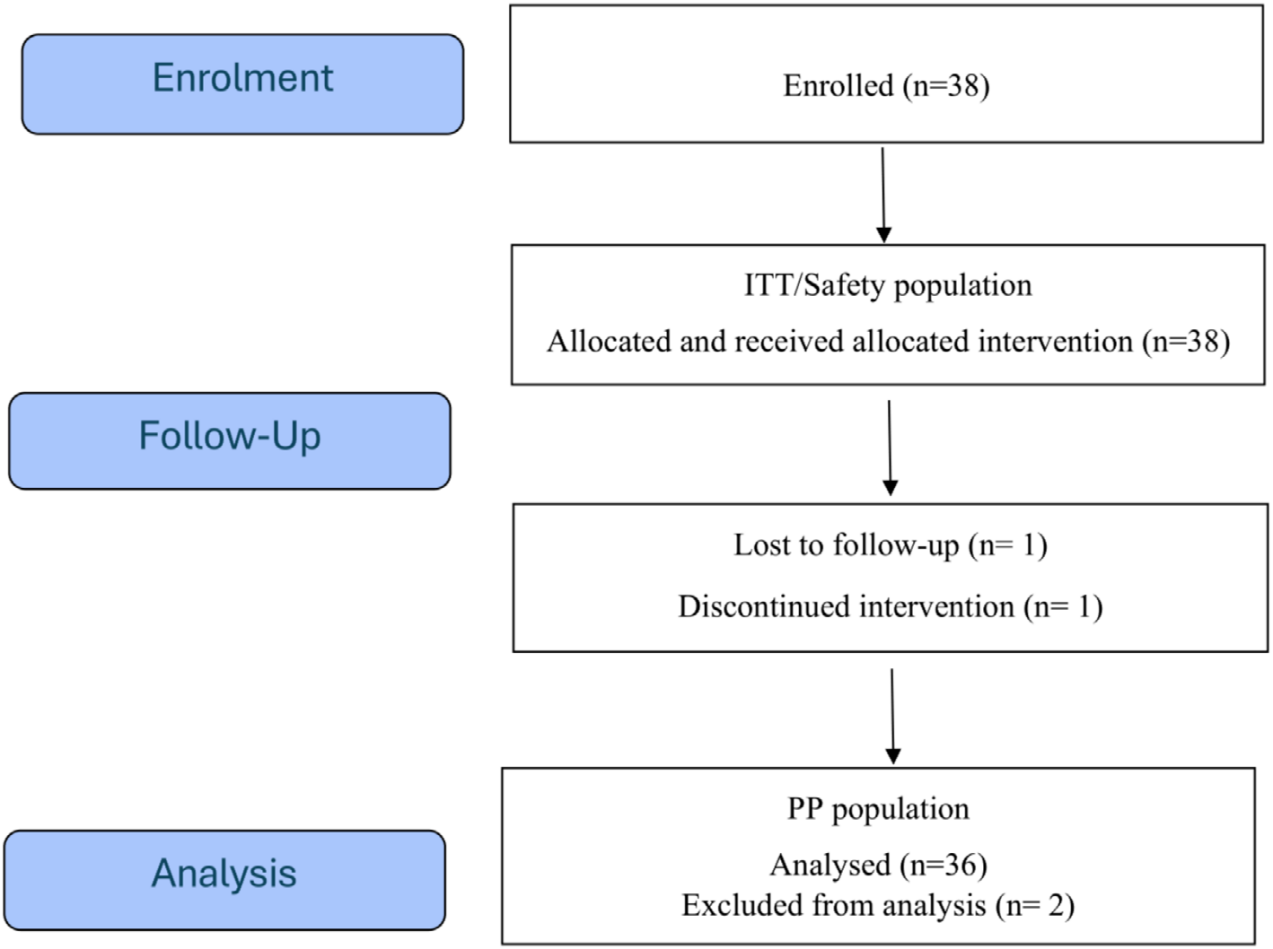
Patient disposition by Consort diagram.

Patients were recruited from 11 November 2022 (First Patient First Visit) to 22 September 2023 (Last Patient Last Visit). Thirty‐eight patients with acne vulgaris were considered eligible to complete the study and were included in the intention to treat (ITT) and safety populations, which coincided, and enrolled to receive the MDIC. Thirty‐six patients completed the trial; two patients were excluded from the Per‐Protocol (PP) analysis (1 lost‐to‐follow‐up and 1 discontinued the intervention; both cases were due for personal reasons, not related to safety). The patient disposition is graphically displayed in Figure 1.

The tested MDIC gel was self‐administered by the patients at home for the entire duration of the study and the application was as indicated in the IFU, “several times a day as needed”. The study population applied MDIC gel a minimum of two to a maximum of three times per day.

Demographics and baseline characteristics are shown in Table 1. Baseline characteristics were similar between sexes, except for age (years), where a statistically significant difference was observed (*p* = 0.035), males being younger.

### 4.2 Primary Outcome Analysis

The decrease from day 0 to day 56 (end of the treatment) in the total number of lesions present on the body area mainly interested in the disease, which was the primary outcome to assess the performance of the tested medical device, is shown in Table 2.

**TABLE 2:** Change in the total number of lesions at 28–56 days after the initiation of treatment, compared to baseline.

	Visit day 0	Visit day 28	Visit day 56
*N*	36	36	36
Mean (SD)	15.78 (16.76)	9.61 (8.01)	6.89 (6.31)
Median	11	7	4.5
Range	[5–102]	[2–30]	[0–25]

*Note:* Day 0 versus day 56: *p* < 0.001 (Wilcoxon test). Day 0 versus day 28: *p* < 0.001 (Wilcoxon test).

**FIGURE 2:**
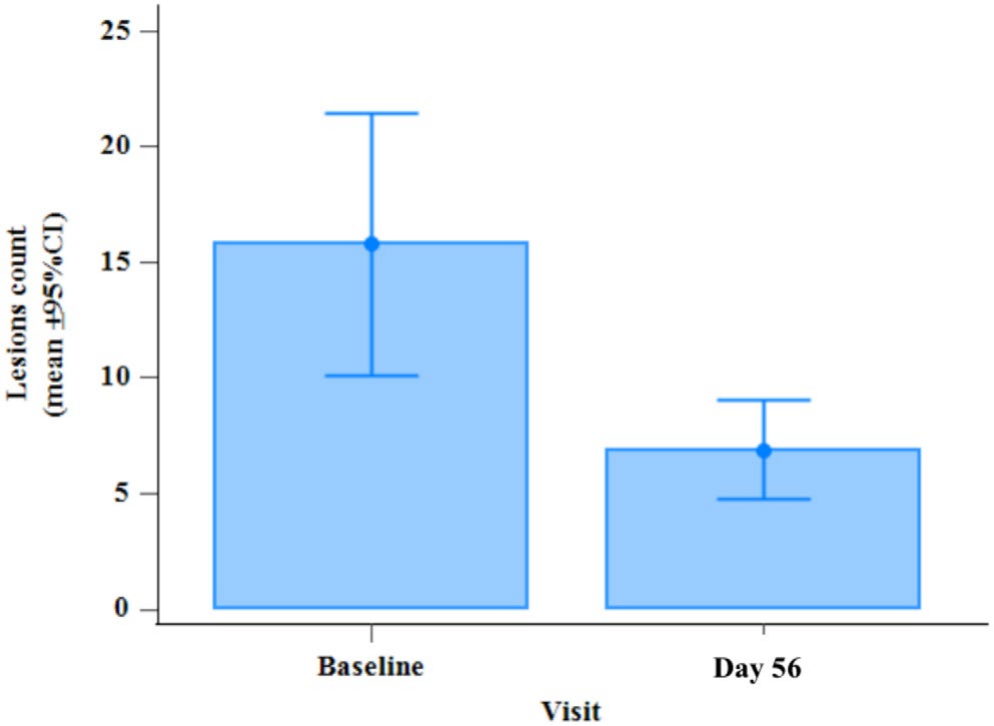
Bar chart of the total number of lesions at day 56 visit, compared to baseline (*p* < 0.001, with Wilcoxon signed rank test).

A 2‐fold decrease in the number of lesions between baseline (day 0) and final visit (day 56) visits, highly statistically significant (−56.3%, *p* < 0.001), was evidenced (Wilcoxon signed rank test) (Figure 2).

A decrease in the number of lesions between day 0 and day 28 was also observed (−39%, so statistically significant with *p* < 0.001).

### 4.3 Secondary Outcomes Analysis

The results of GAGS score at 28 and 56 days and compared with baseline values, assessed by the Investigator, were summarized in Table 3.

**TABLE 3:** Global Acne Grading System (GAGS) Score at day 0 compared to day 28 and day 56.

	Visit day 0	Visit day 28	Visit day 56
*N*	36	36	36
Mean (SD)	13.83 (4.87)	9.58 (4.02)	7.03 (3.68)
Median	13	9	7.5
Range	[6–26]	[3–19]	[0–14]

*Note:* Day 0 versus day 28 versus day 56: *p* = 0.048 at Friedmann test. Day 0 versus day 28: *p* = 0.001 at post hoc Durbin‐Conover pairwise Wilcoxon signed‐rank test. Day 0 versus day 56: *p* < 0.001 at post hoc Durbin‐Conover pairwise Wilcoxon signed‐rank test. Day 28 versus day 56: *p* = 0.048 at post hoc Durbin‐Conover pairwise Wilcoxon signed‐rank tests.

A highly statistically significant reduction in acne scores observed from baseline to day 28 (−30.7%, *p* = 0.001) and day 56 (−49.2%, *p* < 0.001) is shown. Also, an improvement of the GAGS score between day 28 and day 56 (−26.6%, *p* = 0.048) has been shown.

**FIGURE 3:**
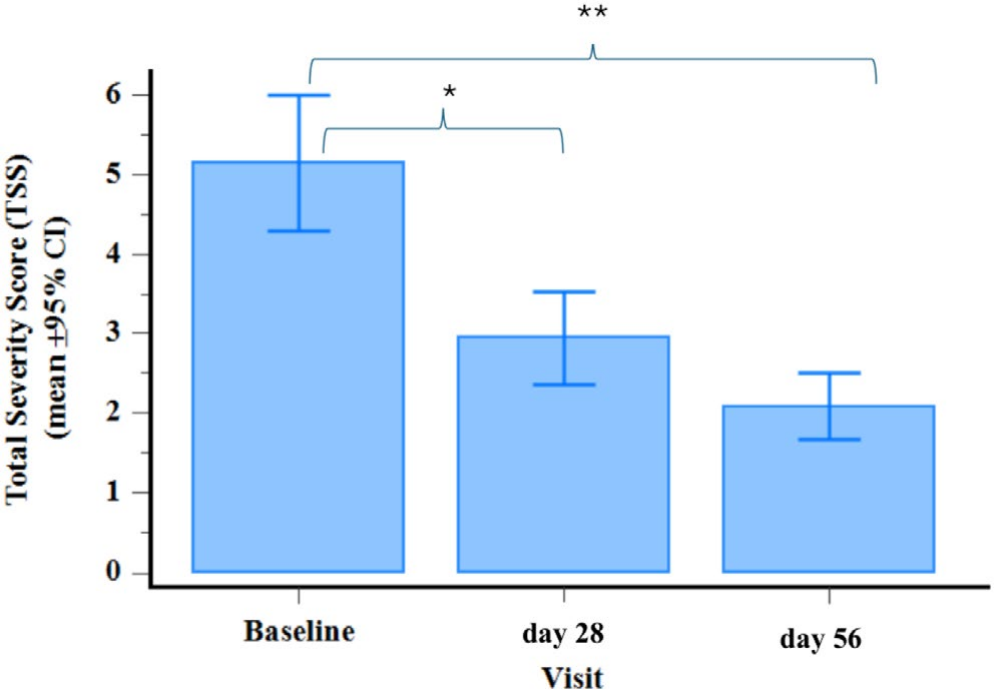
Bar chart with changing in Total Severity Score (TSS) from baseline to the final day 56 visit (day 0 vs. day 28 vs. day 56) *p* < 0.001 at Friedmann test. *Day 0 versus day 28 *p* = 0.002 at post hoc Durbin‐Conover pairwise Wilcoxon signed‐rank test. **Day 0 versus day 56 *p* < 0.001 at post hoc Durbin‐Conover pairwise Wilcoxon signed‐rank test.

The Investigator assessed the change in TSS between the baseline and day 28 and day 56 (Figure 3). The reductions were statistically significant from baseline to both day 28 (− 42.8%, *p* = 0.002) and day 56 (− 59.5%, *p* < 0.001), indicating a substantial improvement in acne vulgaris severity over the 56‐day treatment period. No significant difference was observed in the TSS between day 28 and day 56 (−29.3%, *p* = 0.442).

**FIGURE 4:**
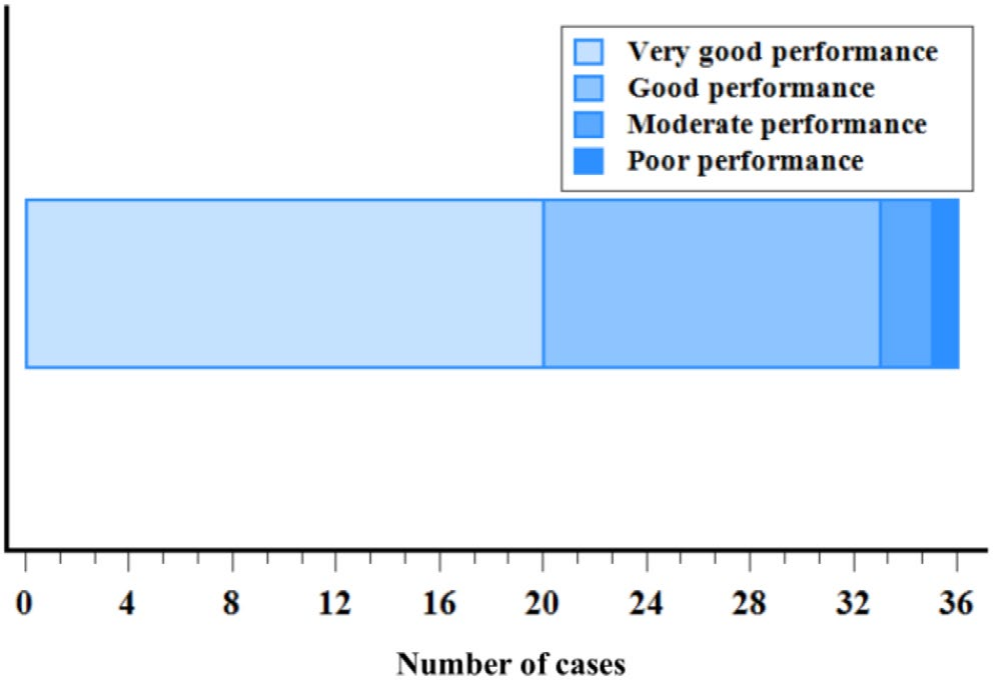
Investigator Global Assessment of Performance (IGAP) assessed by photographs at the final visit (day 56).

**FIGURE 5:**
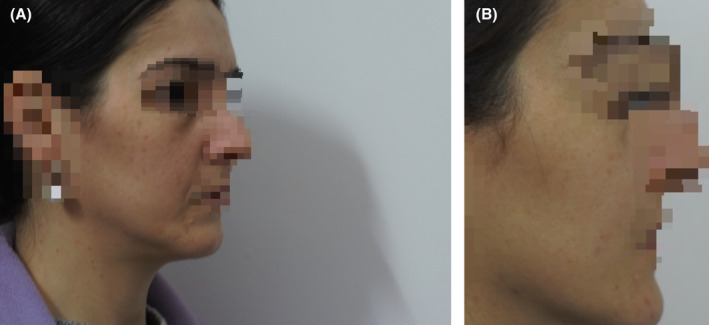
(A) Photograph at baseline visit of a subject include in the study. (B) Photograph at day 56 of the same subject of Figure 5. The photographs were taken at each visit of the study and evaluated by a blind Investigator for performing the Global Assessment of Performance (IGAP).

Investigator Global Assessment of Performance (IGAP) was assessed by means of photographs taken by the Investigator at each visit of the study. Figure 4 summarizes the evaluation performed by a blind Investigator of IGAP comparing the photographs taken at baseline and the final visit (day 56). According to a 4‐point scale, very good performance was indicated for 55.6% of subjects, good performance for 36.1%, and moderate performance for 5.6%. The blind Investigator scored poor performance for only 1 patient out of 30 (2.8%). Figure 5 shows the photographs of a patient at the different visits.

The patient evaluation of the DLQI score, at the end of the treatment period, is summarized in Table 4. The DLQI evidenced a statistically significant difference between the total score on day 0 and on day 56 (− 78.6%; *p* < 0.001). The mean values decreased from 7.53 (SD = 5.62) to 1.61 (SD = 1.74).

**TABLE 4:** Change in the Dermatology Life Quality Index (DLQI) total score at 56 days after the initiation of treatment, compared to baseline.

	Visit day 0	Visit day 56
No effect at all on patient's life	4 (11.1%)	20 (55.6%)
Small effect on patient's life	11 (30.6%)	15 (41.6%)
Moderate effect on patient's life	12 (33.3%)	1 (2.8%)
Large effect on patient's life	0 (0.0%)	0 (0.0%)
Very large effect on patient's life	8 (22.2%)	0 (0.0%)
Extremely large effect on patient's life	1 (2.8%)	0 (0.0%)

*Note:* The *p* < 0.001 by Bhapkar test for marginal homogeneity.

The Bhapkar test showed a statistically significant difference between the visits (*p* < 0.001), as smaller effects on the patient's quality of life were recorded at the final visit. The −78.6% reduction between day 0 and day 56 (final visit) was also confirmed by the Wilcoxon signed‐rank test (*p* < 0.001).

The great majority of the patients reported a high degree of satisfaction with the received treatment, filling the Treatment Satisfaction Questionnaire at the end of treatment on day 56 (63.9% very satisfied and 8.3% satisfied). Ten patients (27.8%) were moderately satisfied.

### 4.4 Safety Analysis

An overall assessment of safety was done by means of the IGAS and PGAS evaluated at day 56. The safety was judged as “very good” and “good” in 80.6% and 19.4% of cases, respectively, at day 56 by the Investigator, and as “very good” and “good” in 75.0% and 25.0% of cases, respectively, at day 56 by patients.

Overall, three patients reported at least 1 adverse event (AE) during the study; eight AEs were reported in total (1 patient reported 1 AE, 1 patient reported 2 AEs, and 1 patient reported 5 AEs). All AEs were resolved by the end of the study; all were judged mild and related to the MDIC. All AEs were application‐site reactions (such as irritation and 6 cases of pruritus). No serious adverse events (SAEs) occurred during the trial, and neither any ADE nor DD were reported during the study period.

## 5 Discussion

Collecting real‐world data (RWD) on MDIC gel was the goal of our study: therefore, we chose the improvement in the total number of lesions on the affected body area as the primary outcome. In fact, in clinical practice, this is the change that is constantly monitored by every physician and immediately indicated by patients during the treatment period. In addition, total lesion count is a very commonly reported outcome in acne clinical trials and allows for an indirect comparison with other products tested in the literature for the treatment of mild to moderate acne (the same severity as in our study). MDIC demonstrated a 56% reduction in total lesion count after 8 weeks of treatment in our study. Comparison with BPO, the direct benchmark of MDIC, showed similar results: a 57% reduction was reported after the administration of 4% BPO for 8 weeks [10] and 5% BPO for 12 weeks [23]. On the other hand [24], a 44% reduction was reported sing a 10% BPO for 8 weeks. The improvements observed in the efficacy parameters examined in our study confirmed the results reported by Milani [10] and Veraldi [11] in two trials, in which the efficacy of BPO and HPO treatments was found to be comparable. However, it is noted that the populations enrolled in the Milani [10] and Veraldi [11] studies were affected by a higher degree of acne severity than that observed in our study. In fact, after 8 weeks of HPO treatment, the total number of lesions observed in the Milani [10] study decreased from 35 ± 8 to 16 ± 8, while our trial showed a reduction from 15.78 ± 16.76 at baseline to a result of 6.89 ± 6.31 at the end of treatment. Similarly, in the Veraldi study [11], GAGS decreased from 16.0 ± 1.2 at baseline to 12.0 ± 1.1 at day 56; in our trial, the decrease was from a baseline of 13.83 ± 4.81 to 7.03 ± 3.68.

In the present study, safety was very good, and the number of subjects experiencing AEs (always mild and transient) was consistent with the data reported by Milani [10] in the population treated with HPO: two of thirty (6.6%) in Milani [10] and three of thirty‐eight (7.8%) in our study. In addition, the majority of AEs in our study were application site reactions, and there were no cases of photosensitisation (the most common AE following retinoid administration), even in the treated population during the summer study period (from June 2023 to September 2023). These data confirmed previous in vitro predictive models of phototoxicity showing that MDIC gel is not phototoxic/photo‐irritant, can be considered nonsensitizing (delayed‐type hypersensitivity test), is not irritating at the skin level (skin irritation test) and does not cause any potential sensitizing effects [25]. Both the reduction in the total number of lesions at the 8‐week follow‐up (this is the sign always reported by patients in the dermatology clinic) and the optimal safety (100% excellent to very excellent) during the 8‐week course are probably related to the optimal improvement in the quality of life demonstrated by the patients enrolled in this study. The tool used to measure QoL in our study was the DLQI: this choice was due to the need to have in the study the same setting of the standard dermatology practice of the participating centers. In fact, the DLQI is a 10‐item questionnaire that is self‐explanatory, easy to complete, and takes only 2 min for the patient to complete. This extreme simplicity obviously gave the risk of neglecting or even losing some elements able to affect the quality of life of the patient with acne. In fact, comparing the DLQI with the most recently developed questionnaire for acne [26], the absence of lack of self‐confidence, stress, suicidal thoughts, and loneliness is evident. Several studies have reported that men and women with acne perceive their QoL very differently [27]. In our study, the number of participants by gender (29 females and only 7 males) did not allow us to detect significant differences. Finally, the very positive feedback of the patients regarding the treatment administered in our study was evidenced by the statistically significant difference between the DLQI scores on day 0 and day 56 for all 10 items (and of course for the total score).

Our study has several potential limitations. First, the selection of a population affected only by mild to moderate acne severitywas justified by the fact that we were conducting a PMCF study and, according to the regulatory requirements for this type of study, the enrolled patients should be treated with the same dosing regimen as outlined in the IFU for the medical device being tested. Thus, we did not intend to conduct a randomized clinical trial (RCT), which could only include severe cases from an over‐selected population. Rather, our intention was to provide a real‐life snapshot of the routine dermatology practice carried out by specialist physicians in their outpatient clinics, reporting the safety and potential improvement in efficacy when administering the medical device MDIC gel to patients with mild to moderate acne. Accordingly, the same type of examination typically performed by a physician in an outpatient setting and the same posology indicated in the IFU were applied to a sample of the typical population seen in a dermatology outpatient clinic. On the other hand, this flexible dosing frequency, depending on the skin condition and extent of the treated areas, was consistent with the inclusion criteria of the study, which considered a fairly wide range of acne severity (from mild to moderate). In addition, this attitude followed the real needs of the treatment, avoiding the rigidity of a fixed regimen, which can find its use of choice in RCTs where inclusion criteria are strictly defined.

A second weakness of the study was the open‐label, not comparative, design with the lack of a control or comparator group (e.g., benzoyl peroxide or hydrogen peroxide without glycine) and the nonadoption of a split‐face design that would have allowed us to make a direct comparison between the treated and untreated sides of the face. This simple technique is usually used in RCTs with optimal performance and is also well accepted by patients, since at the end of the study their untreated side of the face receives the treatment with the tested product. Once again, the justification for this limitation was the regulatory requirement of the present study: it was a PMCF study where it was mandatory to follow the standard clinical practice of the participating center, which obviously does not have in its standard procedure the use of a comparator group nor the split‐face technique. In addition, the Ethics Committee would have rejected the study that claimed to follow the IFU of the tested medical device in a setting of standard dermatology practice and that used a typical RCT experimental design. We planned to use the efficacy data collected in this study to calculate the sample size for a future prospective large trial with MDIC. Similarly, as this was the first study to collect RWD on MDIC gel, we decided to limit the sample size of the study to the number strictly necessary to ensure the collection of efficacy data that will be essential for sample size calculation in a future prospective pragmatic large trial.

We did not collect data on the diet of the enrolled patients during the 8 weeks of treatment. This is an additional limitation of our study, as interesting considerations could be made, particularly regarding the correlation between QoL and consumption of chocolate, sweets, oily foods, and dairy products, which several studies have found to be significantly associated with acne [28].

Finally, there are limitations regarding photos. All included patients gave their consent to be photographed in the study, but only a limited number also agreed to have their anonymized images published in medical journals. This is obviously an issue, even if this ethical clause guarantees the respect of the patients' willingness and privacy. Another limitation related to the photographs was that in the Clinical Photography Procedures of the study it was reported that if the lesions were not only on the face and the visibility from 0° and 45° was poor compared to the front of the face, the Investigator could take the photographs properly according to his/her opinion. This freedom of choice was suggested to concentrate the focus of the photo where the acne lesions were more evident, thus optimizing the blinded evaluation. The bad side of this guidance was that in several cases the photographs of two consecutive visits seemed to be taken from different angles and lighting conditions.

A recent review [29] showed that the second effective acne treatment, just after oral isotretinoin, was BPO administered in triple therapy with topical antibiotic and topical retinoid. As a consequence, it is likely that combination therapy will remain the standard of care for the majority of acne patients, at least in the near future, until novel therapies capable of targeting the pathogenicity of acne lesions with minimal side effects become available [30]. On the other hand, the weak ring of BPO with topical antibiotic and retinoid combination therapy is given by the presence of its known side effects (irritation, staining, allergy) [31]. For this reason, the use of new products such as MDIC in the substitution of BPO and with similar efficacy may represent a valid alternative for dermatologists in the treatment of acne. In our opinion, the positive results in terms of reduction in total lesion count, reduction in GAGS and TSS scores, improvement in IGAP and DLQI scores, and Treatment Satisfaction Questionnaire clearly demonstrate that the 8‐week administration of MDIC gel produced a clinical benefit in the subjects treated in this study. In addition, the optimal safety and compliance of the tested medical device are fundamental requirements for a product to be administered over a long period of time, which could potentially be combined with other treatments, such as retinoids, as first‐line treatment for mild to moderate acne.

## Conflicts of Interest

Dionisio Franco Barattini is employed at Opera CRO, the contract research organization that managed the study. Luca Barattini is a former intern at TIGERMED Italy Srl, the company involved in data management and statistical analysis. Luca Stucchi is employed at BMG Pharma SpA, the Sponsor of the study. Meda‐Elena Stefancu, Ionel Botnaru, and Carmen Vizman declare no conflicts of interest. The funder BMG Pharma SpA, Milano, Italy, had no role in the design of the study; in the collection, analyses, or interpretation of data; in the writing of the manuscript; or in the decision to publish the results.

## Supporting information


Data S1.

## Data Availability

The full protocol of the study and the data sets analyzed are available from the corresponding author upon request.
