# Supplementary material for: Performance and Safety of the Medical Device Ialuxid Gel in the Treatment of Mild–Moderate Acne Vulgaris: An Open‐Label, Noncomparative Multicentre Interventional Clinical Trial
Source: J Cosmet Dermatol. 2025 Mar 3;24(3):e70084. doi: 10.1111/jocd.70084 (PMC11875049; doi:10.1111/jocd.70084)
Supplement: Supplementary file 1 — Data S1. [file JOCD-24-e70084-s001.pdf]

Clinical Photography Procedures in the study protocol OPBMG/0122/MD

Purpose

To give indications to the people (Investigators, Co-Investigators, CRO personnel) involved in taking and managing photographs to the patients in the study OPBMG/0122/MD to perform this tasks in a safe, confidential, technically and clinically correct way.

Informed consent and confidentiality

Patients enrolled in the OPBMG/0122/MD study must have signed the informed consent form and GDPR statement as required by the study protocol.

One of the study outcomes is the Investigator Global Assessment of Performance (IGAP), which is assessed by photographs taken by the investigator at each study visit. For this reason, each patient will also be required to read and sign a special consent form to be photographed. This form will allow the investigator to evaluate the patient's performance based on his/her images. Signing this consent form will be mandatory for inclusion in the OPBMG/0122/MD study.

An additional consent form may be signed by the patient to allow the use of his/her image for publication in medical journals.

This form will allow the investigators to submit to a medical journal or present at a medical congress the anonymized images of patients who have given their consent. Signing this consent form will be optional and not mandatory in the OPBMG/0122/MD study.

Timing

Standardized facial photographs of each patient will be taken at baseline and at weeks 2, 4, and 6 visits to provide photographic documentation of the observed changes in the treated area(s) and will be used by the investigators for clinical grading assessments.

Before making photographs

Before starting the photo procedure, the patients will be instructed to clean their faces, remove all makeup and jewellery. In addition, the investigator will suggest to the patient that he or she wear clothes appropriate for the temperature of the room in which the photographs will be taken, in order to avoid sweating or chills.

Performing the photographs

The Investigator will take care of the following:

- Pose
  - ✓ Center the patient's head in the photograph
  - ✓ Ask the patient to look directly at the camera
  - ✓ Make sure there is no hair in the patient's face or eyes
  - ✓ Background must be white (wall or paper background accepted)
  - ✓ Make sure the patient's facial features are clearly visible from the bottom of the chin to the top of the head.
  - ✓ Make sure the patient isn't tilting their head up/down or left/right.
- Expression
  - ✓ Ensure that the patient maintains a neutral facial expression (no raised eyebrows, frowns).

  - ✓ Ensure patient keeps both eyes open and mouth closed.
  - ✓ Make sure the patient is not smiling
- Glasses, head covers
  - ✓ Ensure that the patient is not wearing any type of glasses.
  - ✓ Make sure the patient is not wearing a head covering, unless it's for religious reasons.
  - ✓ Make sure the patient is not wearing a hair band.
- Technical Characteristics (camera, lens, focus, and light)
  - ✓ A Nikon D300(s)/Digital camera body and an AF 18-55mm lens will be used for all images.
  - ✓ The lens will always be set at the maximum focal length of 55mm. (Digital images (RAW; 12 bits per channel) will be captured using Nikon Capture software.
  - ✓ A special ring light (Ring 48 Macro RingLite LED) will be attached to the lens and all images will be taken using only the artificial light from the ring light.
  - ✓ Photos must be in focus and correctly exposed (at 55mm focal length, with ring light as light source).
- Characteristics of the Pictures
  - ✓ Photos must be taken at the highest resolution in RAW format.
  - ✓ Digital full face photographs.
  - ✓ Right and left sides of the patient's face (at a 45° angle to the side of the face identified by the examiner) in one pose for each side.
  - ✓ If the lesions are not only on the face and visibility from 0° and 45° is poor compared to the front of the face, the photograph will be taken properly according to the opinion of the investigator.
  - ✓ A third pose looking directly at the camera for a frontal view (0° angle).

Post-processing procedures

The only post-processing allowed is the anonymization of all images. This activity will be performed by a blinded person of the CRO by covering the patient's eyes and, if necessary, cropping the untreated facial areas (e.g., chin, lips). Only after the anonymization process is complete, the images will be sent to the sponsor via transfer secure.

Once the photograph/image has been uploaded to the patient's record and viewed from within that record, the original image can be securely deleted. The photograph/image will then become part of that patient's record and will be retained for the same period of time after the trial is completed.

References

- Gottfried Lemperle, M.D., Ph.D., Ralph E. Holmes, M.D., Steven R. Cohen, M.D., and Stefan M. Lemperle, M.D., A Classification of Facial Wrinkles, Division of Plastic Surgery, University of California, and FACESplus, Inc. Received for publication September 15, 2000; revised January 5, 2001
- Jiang L.I., Stephens T.J., Goodman R, SWIRL, a clinically validated, objective, and quantitative method for facial wrinkle assessment, Thomas J. Stephens & Associates Inc., Carrollton, TX, USA
- Stephens T, Sigler M, Herndon J, Dispensa L, Le Moigne A, A placebo-controlled, double-blind clinical trial to evaluate the efficacy of Imedeen® Time Perfection® for improving the appearance of photodamaged skin, Clinical, Cosmetic and Investigational Dermatology, 15 March 2016 Volume 2016:9 Pages 63—70
